# The Institutional Worker

**Published:** 1912-06-29

**Authors:** 


THE
### INSTITUTIONAL WORKER
**Being a Special Supplement to "The Hospital"**

### Editor's Notices.

**Contributions:**
Contributions should be written, or preferably typed, on one side of the paper only, and all articles sent in are accepted upon the distinct understanding that they are forwarded to The Hospital only.

The Editor cannot undertake to return MSS. not used, but when a stamped directed envelope is enclosed the MSS. may be returned if a special request is made.

Accepted articles and paragraphs of news will be paid for after publication at the scale rate.

**Address:**
To prevent delay all contributions and letters on editorial business must be addressed exclusively to the Editor, "The Hospital," 29 Southampton Street, Strand, London, W.C. It is important that this regulation shall be strictly observed.

**Correspondence:**
Correspondence on all subjects is invited. The name and address of correspondents must be given as a guarantee of good faith, but not necessarily for publication.

**Special Articles:**
Special articles are invited, and questions, inquiries, and paragraphs upon all matters relating to the work, administration, and management of general, special, mental, fever, cottage and convalescent hospitals, sanatoria, homes, institutions, societies and organisations for the treatment or care of the sick, injured and dependants of all classes. Every matter affecting the interests and welfare of the staff of all grades working in these institutions will receive special consideration.

**Administrative Medicine, Research and Items of News:**
Special terms will be paid for approved articles on subjects in this wide field by experts who have made some section of it their special study and interest. We wish to give prominence to every movement tending to advance accuracy of diagnosis, efficiency in treatment, and the development of modern methods to eradicate disease and promote the welfare of the suffering.

**A Bureau of Information:**
We invite inquiries and applications for information and help in providing for that numerous class of sufferers who require special care or house-room which they cannot obtain in their own homes or secure for themselves. **We cannot, however, prescribe, or recommend practitioners.**

**Books for Review:**
Publishers are particularly requested to send advance proofs of any new books of importance whenever possible, as the Editor has made arrangements to publish immediate reviews on a new plan.

**Photographs, Plans, Blocks, and Illustrations:**
It is requested that wherever possible MSS. may be accompanied by illustrations in any of the above forms. The name of the sender and of the article to which it belongs should be written on the back of each photograph, drawing, or block for purposes of identification.

**Local Papers:**
Newspapers containing reports or news paragraphs **should be marked** and addressed to the Sub-Editor.

**The Coupon System:**
A coupon will be found at the bottom of the third inside page of the cover each week. This coupon must be attached to every question or inquiry to which an answer is desired in The Hospital.

**ARE YOU ACQUAINTED WITH**

**"THE HOSPITAL"**

**Bureau of Information?**

**This departure is a practical help to every Medical and Institutional Worker, Health Visitor, District Nurse, and all interested in and associated with the prevention of Disease, alleviation of Sickness, and the advancement of National Health.**

**ENQUIRIES WELCOMED ON EVERY SUBJECT.**

See page 334 for further particulars.

### Manager's Notices.

**All letters on business matters must be addressed to The Manager, "The Hospital," 28 and 29 Southampton Street, London, W.C.**

**Advertisements:**
To ensure insertion, all advertisements for The Hospital must reach the Manager not later than Wednesday morning in each week. For scale of charges see pages v and 2.

**Subscriptions,** which may commence at any time, are payable in advance. Cheques and Post-Office Orders should be crossed London County and Westminster Bank, Covent Garden Branch, and made payable to the Manager of The Hospital.

Rates of Subscription (including Postage).

| *United Kingdom.* | | *Foreign and Colonial.* | |
|---|---|---|---|
| Three Months ... | ... 2s. 0d. | Three Months ... | ... 2s. 6d. |
| Six Months ... | ... 4s. 0d. | Six Months ... | ... 5s. 0d. |
| Twelve Months ... | ... 6s. 6d. | Twelve Months ... | ... 8s. 8d. |

*SUBSCRIPTION FORM.*

*Please send me* The Hospital *for*..................*months, commencing with your issue of*............................., *for which please find enclosed*...........................

*Name*..................................................

*Address* ..................................................

..................................................

*Date*..................................

*To* .................................... *Newsagent.*

**Remittances:**
**It is especially requested that remittances be made by Cheque or Postal Order, and in halfpenny stamps only when the amount is under sixpence.**

